# The Whole Transcriptome Involved in Denervated Muscle Atrophy Following Peripheral Nerve Injury

**DOI:** 10.3389/fnmol.2018.00069

**Published:** 2018-03-07

**Authors:** Jian Weng, Peixun Zhang, Xiaofeng Yin, Baoguo Jiang

**Affiliations:** Department of Orthopedics and Trauma, Peking University People’s Hospital, Beijing, China

**Keywords:** denervated muscle atrophy, sequencing, whole transcriptome, ncRNAs, peripheral nerve injury

## Abstract

Peripheral nerve injury (PNI) usually leads to progressive muscle atrophy and poor functional recovery. Previous studies have demonstrated that non-coding ribonucleic acid (ncRNA) is a key regulator of muscle atrophy and beneficial for the treatment of PNI. We aimed to analyze the whole transcriptome involved in denervated muscle atrophy after PNI. Animal models of sciatic nerve injury were assessed at 0 (control group), 1, 2, 4, and 8 weeks after injury. The expression patterns in the whole transcriptome in the gastrocnemius muscle were profiled using RNA sequencing at each time point and compared to that obtained in the control group. Six-hundred and sixty-four long non-coding RNAs, 671 microRNAs, 236 circular RNAs, and 12,768 messenger RNAs (mRNAs) were differentially expressed (DE) after injury. Changes in some of the DE ncRNAs and mRNAs were validated using quantitative polymerase chain reaction. Gene Ontology and Kyoko Encyclopedia of Genes and Genomes analysis revealed the potential functions of and relationships among the DE ncRNAs and mRNAs. To our knowledge, this is the first study to expound the whole transcriptome involved in denervated muscle atrophy, and provides a theoretical basis for further research targeting ncRNAs.

## Introduction

Peripheral nerve injury (PNI) may result in developmental atrophy of the target skeletal muscle and poor functional recovery due to delayed surgery. Due to their slow rate of regeneration (1–3 mm/d), the injured axons usually require at least 3 months to regenerate to distal organs. However, the target muscle usually undergoes atrophy and cannot be reinnervated after the prolonged period required for axonal regeneration. This in turn leads to poor neurological functional outcomes (Höke and Brushart, 2010). The major effects of progression of this type of atrophy are a loss in muscle mass and decreased strength. In order to improve functional motor recovery after nerve injury, it is necessary to reduce the time required for innervation and to decelerate the progress of denervated muscle atrophy (Moimas et al., 2013). The progression of denervated muscle atrophy includes a series of pathological events and complex molecular mechanisms that are still largely unknown. Therefore, investigation of the underlying mechanisms of denervated muscular atrophy and the search for new therapeutics for this condition are particularly important. Our team has studied peripheral nerve repair and the protection of atrophic muscles for decades. The transcriptome has become an important field of study in the post-genomic era, and has unique advantages in the study of PNI (Phay et al., 2015). We therefore have great interest in the whole transcriptome involved in denervated muscle atrophy. Our aim is to better understand the genetic and neurobiological bases of denervated muscle atrophy in order to develop effective therapeutic strategies for this condition.

Significant molecular changes occur in the target muscle before it is reached by the regenerated axons (Sando and Cederna, 2016). Previous studies have shown that non-coding ribonucleic acids (ncRNA) plays a key role in regulating protein expression during muscle atrophy (Wilkins et al., 2004; Wang et al., 2011). In contrast to typical RNA, ncRNAs do not encode proteins, but instead functionally regulate the expression of proteins (Mattick and Makunin, 2006). ncRNAs may be subdivided based on length into small ncRNAs (<200 bp), which include long non-coding RNAs (lncRNAs) and microRNAs (miRNAs), and circular RNAs (circRNAs) >200 bp (Thum, 2014). The functional mechanisms of action of ncRNAs in denervated muscular atrophy have not been fully studied. To date, few cases have been reported in this field. Therefore, a systematic analysis of the expression of ncRNAs involved in the progression of denervated muscular atrophy is essential for the exploration of effective treatments and the prevention of this disorder. The transcriptome has a dynamic characteristic with highly specific features in space and time. Furthermore, the whole transcriptome is known to be a dynamic reflection of changes in ncRNAs and messenger RNAs (mRNAs). This can be used to further study the mechanisms underlying denervated muscle atrophy. Next-generation RNA sequencing (RNA-seq) is an extremely sensitive method for analyzing differentially expressed (DE) RNAs (Sîrbu et al., 2012). RNA-seq is superior to the use of gene microarrays, as it is not limited to only a subset of known RNAs, but can also be used to analyze the entire transcriptome in tissues and organs to provide biological information regarding possible functions of annotated or novel genes (Hurd and Nelson, 2009).

Here we first evaluated the expression profiles of ncRNAs and mRNAs in denervated muscle atrophy after PNI using RNA-seq. We detected significant expression changes in ncRNAs in the gastrocnemius muscle. Our findings provide a novel information platform that can be used to identify target genes involved in the mechanisms underlying denervated muscle atrophy. In addition, we utilized Gene Ontology (GO) and Kyoko Encyclopedia of Genes and Genomes (KEGG) analysis to identify the biological processes and pathways associated with the DE genes, such as the mitogen-activated protein kinase (MAPK) signaling pathway. These data will serve as useful resources for the development of therapeutic strategies or novel diagnostics in the future.

## Materials and Methods

### Animal Model and Tissue Collection

Fifteen healthy female C57BL/6 mice (weighing 22–25 g and aged 6–8 weeks) were randomly assigned to five experimental groups. Three mice were assigned to each group of mice assessed at 0 (control group), 1, 2, 4, and 8 weeks after sciatic nerve injury. The mice were deeply anesthetized with an intraperitoneal injection of 10% chloral hydrate (3 ml/kg). Once the mice were completely anesthetized, a 0.5 cm-long incision was made over the sciatic nerve of the left hind limb. The sciatic nerves were then exposed by gentle separation and cut approximately 1 cm proximal to the division of the tibial and common peroneal nerves. The incisions were closed using 4–0 sutures. All procedures were performed under sterile conditions. To evaluate the level of atrophy due to the injury, the animals were euthanized 0, 1, 2, 4, and 8 weeks post-injury. The gastrocnemius muscles were harvested from the operated experimental limb and processed for the next step.

### RNA Isolation and Sequencing Library Preparation

Total RNA was isolated using the Trizol (Invitrogen, Carlsbad, CA, United States) according to the protocol. The quality of the purified RNA was assessed using a BioAnalyzer 2100 (Agilent Technologies; Santa Clara, CA, United States). A NanoPhotometer spectrophotometer (Implen; Westlake Village, CA, United States) was used to determine the quantity of purified RNA by measuring the absorbance at 260/280 nm. All of the RNA samples were stored at -80°C and were sequenced by Novogene using the Illumina platform (Beijing, China).

A total of 8 μg of RNA per sample (3 μg for lncRNA and 5 μg for circRNA) was used to generate libraries for lncRNA and circRNA sequencing. After ribosomal RNAs were depleted using the Epicentre Ribo-zero^TM^ rRNA Removal Kit (Epicentre, Madison, WI, United States), the rRNA-depleted RNAs were treated with RNase R (Epicenter) and extracted using Trizol (Invitrogen). Sequencing libraries were generated using the NEBNext^®^ Ultra^TM^ Directional RNA Library Prep Kit for Illumina^®^ (NEB, Ipswich, MA, United States) according to the protocol. First, performed by the divalent cations in NEBNext First Strand Synthesis Reaction Buffer at high temperature. First strand cDNA was synthesized using a random hexamer primer and M-MuLV Reverse Transcriptase (RNase H-). DNA Polymerase I and RNase H were then used to synthesize the second strand cDNA. The deoxythymidine triphosphates in the deoxynucleotide mixture in the reaction buffer were substituted with deoxyuridine triphosphates. The remaining overhangs were then made into blunt ends by exonuclease/polymerase activity. The NEBNext Adaptor with a hairpin loop structure was ligated to prepare for hybridization following the adenylation of the 3′ ends of the DNA fragments. In order to select cDNA fragments 150–200 bp in length, the library fragments were purified using the AMPure XP system (Beckman Coulter; Beverly, CA, United States). After size selection using 3 μl of USER Enzyme (NEB), the cDNA was ligated at 37°C for 15 min and then incubated at 95°C for 5 min. Polymerase chain reaction (PCR) was performed using Phusion High-Fidelity DNA polymerase, Universal PCR primers, and Index (X) Primer.

A total of 3 μg RNA per sample was used to prepare the library for miRNA sequencing. Sequencing libraries were generated using the NEBNext^®^ Multiplex Small RNA Library Prep Set for Illumina^®^ (NEB) according to the manufacturer’s instructions. Index codes were used to attribute sequences to the samples. First, the NEB 3′ SR Adaptor was directly ligated specifically to the 3′ ends of miRNAs. Following the 3′ ligation reaction, some 3′ SR Adaptor remained free. At this time, SR RT Primer was used to hybridize the excess 3′ SR Adaptor. The single-stranded DNA adaptor was then transformed into a double-stranded DNA molecule in order to prevent the formation of adaptor dimers. In the subsequent ligation step, the 5′ SR Adaptor did not ligate double-stranded DNAs, which do not undergo ligation mediated by T4 RNA Ligase 1. The 5′ ends of the adapter were then ligated to the 5′ ends of the miRNAs. M-MuLV Reverse Transcriptase (RNase H-) was used to synthesize the first strand cDNA. PCR amplification was performed using the LongAmp Taq 2X Master Mix, SR Primer for Illumina, and Index (X) Primer. An 8% polyacrylamide gel was used to purify the PCR products (100 V, 80 min), and 8 μL of elution buffer was used to dissolve DNA fragments 140–160 bp in length DNA fragments. Finally, the Agilent Bioanalyzer 2100 system was used to purify the products and evaluate the quality of the library.

### Clustering and Sequencing

Index-coded sample clustering was performed using the TruSeq PE Cluster Kit v3-cBot-HS (Illumina) on a cBot Cluster Generation System following the manufacturer’s protocol. An Illumina HiSeq X platform was used to sequence the library preparations and generate the 125/150 bp paired-end reads and the 50 bp single-end reads when the cluster generation was complete.

### GO and KEGG Enrichment Analysis of DE Genes

Gene Ontology enrichment analysis of the DE genes was performed using the clusterProfiler R package to revise the gene length bias. DE genes with corrected *P*-values less than 0.05 were considered enriched. KEGG is a database resource for exploring high-level functions of biological systems at the molecular level using genome sequencing or other high-throughput experimental technologies. We used the clusterProfiler R package to assess the statistical enrichment of DE genes in KEGG pathways.

### Quantitative Real-Time PCR (qPCR)

The remaining samples in each group were validated using qPCR. First strand cDNA was synthesized using a reverse transcription kit (Abm, San Francisco, CA, United States) following the manufacturer’s protocol. The IQ SYBR green SuperMix reagent (Bio-Rad; Hercules, CA, United States) was then used to amplify the cDNA according to the manufacturer’s instructions. Routine agarose gel electrophoresis and melting-curve analysis were used to assess the specificity of the qPCR. The comparative *C*t method was used to quantify gene expression, and relative values were calculated using 2^-ΔΔC_t_^. The calculations for lncRNA and circRNA expression were consistent with those used for mRNA. The internal control used was β-actin. The expression levels of the miRNAs were normalized to the U6 level in each sample. The sequences of the primers used were ENSMUSG00000087523.1 (forward: CACCGCAGGAAGTGAATCTCT, reverse: AAAGCCACGCCAAAGGCTATA); XLOC_015548 (forward: CAGTTTCACTTGCCCAGAGAGC, reverse: CAAACCCACAGTTCAGAGAAACAC); mmu-miR-34c-5p (RT: GTCGTATCCAGTGCGTTGTCGTGGAGTCGGCAATTGCACTGGATACGACGCAATC, forward: GGGAGGCAGTGTAGTTAGCT, reverse: CAGTGCGTGTCGTGGAGT); mmu-miR-142a-3p (RT: GTCGTATCCAGTGCGTGTCGTGGAGTCGGCAATTGCACTGGATACGACTCCATA, forward: GGGTGTAGTGTTTCCTACTT, reverse: CAGTGCGTGTCGTGGAGT); mmu_circ_0001068 (forward: CTGCAGCTAGAGAATGGCCT, reverse: TCGCTGAATTCCTCAACCACG); novel_circ_0011051 (forward: GGTTTGGCACTCTTTGCACC, reverse: GAGCCCTCTTCTCGGGAACT); Myl4(forward: GAGATGAAGATCACCTACGGGC, reverse: CATCTCTTCAGGCTTGGGTTTG); Myom3 (forward: AGGACTGTCACCTTTCTACGTGTC, reverse: GAGTGGGGCCTGTGTAATGTTT); β-actin (forward: AACAGTCCGCCTAGAAGCAC, reverse: CGTTGACATCCGTAAAGACC); U6 (RT: AACGCTTCACGAATTTGCGT, forward: CTCGCTTCGGCAGCACA, reverse: AACGCTTCACGAATTTGCG).

### Statistical Analysis

All data are expressed as means ± standard errors of the mean. One-way analysis of variance was used to statistically analyze muscle weight. The qPCR results were analyzed using Student’s *t*-tests. *P*-values less than 0.05 were considered statistically significant.

## Results

### General Observation of Muscle Atrophy

**FIGURE 1:**
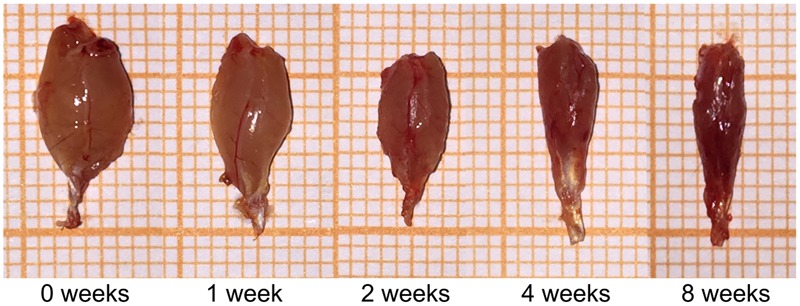
General observation of the gastrocnemius muscle in each group.

The volumes of the gastrocnemius muscle in the injury groups were less than those in the control group after nerve surgery (**Figure 1**). The muscle weight on the experimental side is presented as a percentage of the muscle weight on the control side in each group. This ratio was significantly lower in the surgery groups than in the control group (**Table 1**). The gastrocnemius muscle on the injured side therefore had obvious atrophy after PNI, indicating that our denervated muscle atrophy animal models were successfully established.

**Table 1:** Weights of the gastrocnemius muscle in each group.

	Control 0 weeks	1 week	2 weeks	4 weeks	8 weeks
Wet weight	0.147 ± 0.005	0.074 ± 0.005^∗^	0.062 ± 0.004^∗^	0.052 ± 0.004^∗^	0.048 ± 0.004^∗^
Maintenance rate	100%	50.7%	42.5%	35.6%	32.9%

*n = 5, ^∗^P < 0.05.*

### Quality Control and Correlations of the Data

**FIGURE 2:**
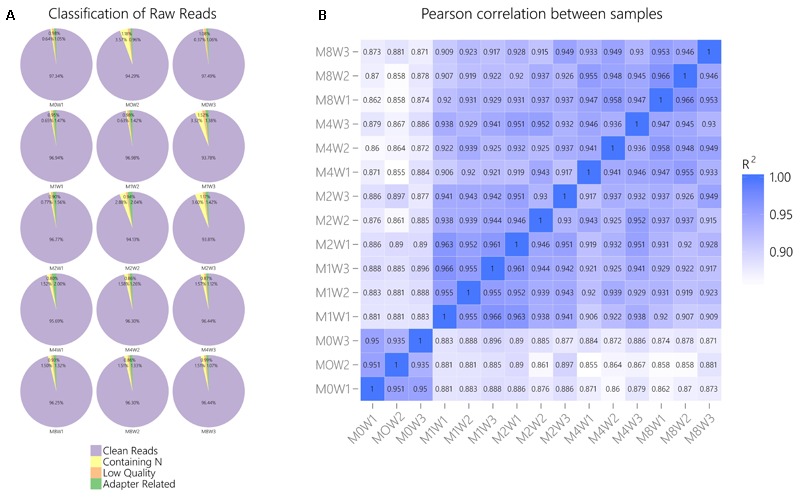
General information regarding the quality of and correlations in the data. **(A)** Clean reads as a percentage of raw reads for each sample. **(B)** Pearson correlation analysis results.

General information regarding RNA-seq data quality was obtained by calculating the ratio of clean data to all data (**Figure 2A**). The ratio of clean reads as a percentage of all raw reads was greater than 93% for all samples. This suggests that the data were high-quality and suitable for subsequent analysis. Correlations were analyzed using the Pearson correlation coefficient, as calculated in R (**Figure 2B**). The *R*^2^ values at each time point were larger than 0.92, suggesting that the biological replicates of the samples in each group met the requirements for subsequent analysis.

### DE ncRNAs and mRNAs After Injury

**FIGURE 3:**
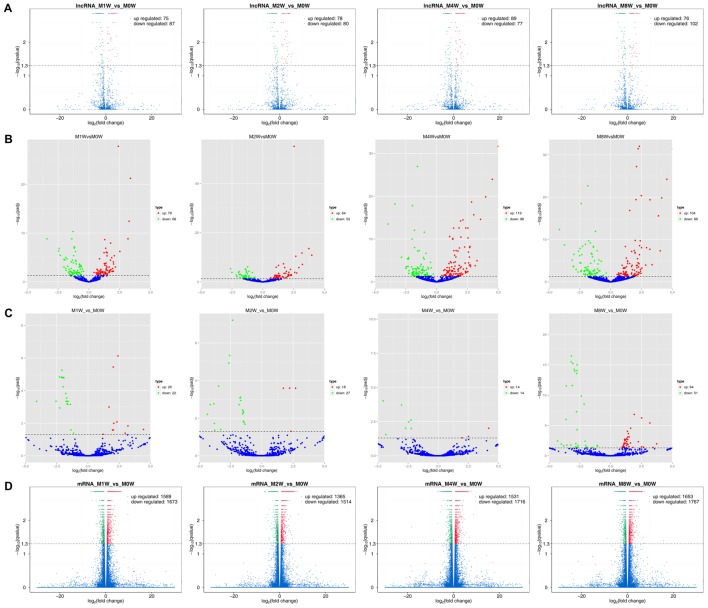
Volcano plots illustrating comparisons of DE ncRNAs and mRNAs between each injury group and the control group. **(A)** DE lncRNAs, **(B)** DE miRNAs, **(C)** DE circRNAs, and **(D)** DE mRNAs.

**FIGURE 4:**
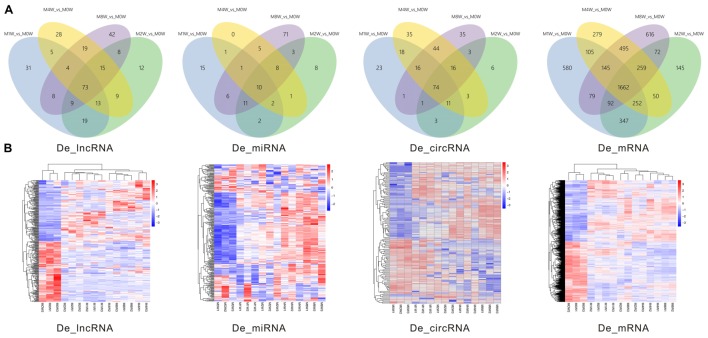
Venn diagrams and heat maps of DE ncRNAs and mRNAs. **(A)** Venn diagrams of the overlap between DE ncRNAs and mRNAs at each time point. **(B)** Heat map displaying the hierarchical clustering of DE ncRNAs and mRNAs at each time point (red indicates up-regulation and blue indicates down-regulation).

**FIGURE 5:**
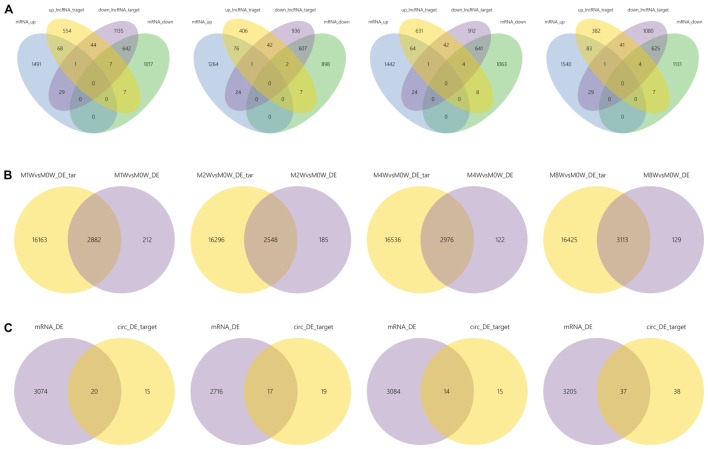
The intersections of ncRNAs and mRNAs at each time point. **(A)** The overlap between target mRNAs of up-regulated lncRNAs, target mRNAs of down-regulated lncRNAs, up-regulated mRNAs, and down-regulated mRNAs. **(B)** Overlap of target mRNAs of DE miRNAs and DE mRNAs. **(C)** Overlap of target mRNAs of DE circRNAs and DE mRNAs.

Differentially expressed ncRNAs and mRNAs at each time point are displayed using Volcano plots, Venn diagrams, and heat maps. Five of the most DE ncRNAs and mRNAs in each injury group compared to the control group are listed in **Tables 2–5**. The Volcano plots, Venn diagrams, and clustering maps for the DE lncRNAs, miRNAs, circRNAs, and mRNAs are shown in **Figures 3, 4**. There were 664 DE lncRNAs (75 upregulated and 87 downregulated at 1 week, 78 upregulated and 80 downregulated at 2 week, 89 upregulated and 77 downregulated at 4 weeks, and 76 upregulated and 102 downregulated at 8 weeks), 671 DE miRNAs (79 upregulated and 68 downregulated at 1 week, 64 upregulated and 53 downregulated at 2 weeks, 119 upregulated and 98 downregulated at 4 weeks, and 104 upregulated and 86 downregulated at 8 weeks), and 236 DE circRNAs (26 upregulated and 22 downregulated at 1 week, 18 upregulated and 27 downregulated at 2 weeks, 14 upregulated and 14 downregulated at 4 weeks, and 64 upregulated and 51 downregulated at 8 weeks) when comparing the injury group to the control group. There were 12,768 DE mRNAs when comparing the injury group to the control group, as follows: 1,549 upregulated and 1,673 downregulated at 1 week, 1,365 upregulated and 1,514 downregulated at 2 weeks, 1,531 upregulated and 1,716 downregulated at 4 weeks, and 1,653 upregulated and 1,767 downregulated at 8 weeks. The intersections of the co-localized or co-expressed target mRNAs with lncRNAs and DE mRNAs, DE miRNAs with DE mRNAs, and DE circRNAs with DE mRNAs are displayed in a Venn diagram (**Figure 5**).

**Table 2:** The top 5 DE lncRNAs at each time point.

Gene id	Gene name	Gene location	INJ FPKM	CON FPKM	Log2 fold change	*p*-value	Time
ENSMUSG00000087410.7	2310065F04Rik	chr11:67112460-67120080	1.65613	24.5852	-3.8919	<0.01	1 w vs. 0 w
ENSMUSG00000085022.2	Gm5860	chr4:82065430-82102808	0.272696	3.82766	-3.8111	<0.01	1 w vs. 0 w
ENSMUSG00000099411.1	2310015D24Rik	chr16:13514130-13521107	0.401347	4.27883	-3.41429	<0.01	1 w vs. 0 w
ENSMUSG00000087523.1	Gm12319	chr11:70654740-70656493	3.04175	149.492	-5.61903	<0.01	1 w vs. 0 w
XLOC_014161	–	chr15:102657086-102660530	0.203383	6.82427	-5.0684	<0.01	1 w vs. 0 w
ENSMUSG00000087410.7	2310065F04Rik	chr11:67112460-67120080	2.72569	24.5852	-3.1731	<0.01	2 w vs. 0 w
ENSMUSG00000000031.15	H19	chr7:142575528-142578143	3436.68	724.059	2.24684	<0.01	2 w vs. 0 w
ENSMUSG00000085022.2	Gm5860	chr4:82065430-82102808	0.540524	3.82766	-2.82403	<0.01	2 w vs. 0 w
ENSMUSG00000099411.1	2310015D24Rik	chr16:13514130-13521107	0.592203	4.27883	-2.85305	<0.01	2 w vs. 0 w
ENSMUSG00000087523.1	Gm12319	chr11:70654740-70656493	5.62741	149.492	-4.73146	<0.01	2 w vs. 0 w
ENSMUSG00000087410.7	2310065F04Rik	chr11:67112460-67120080	2.43621	24.5852	-3.33508	<0.01	4 w vs. 0 w
ENSMUSG00000000031.15	H19	chr7:142575528-142578143	3909.45	724.059	2.43279	<0.01	4 w vs. 0 w
ENSMUSG00000085022.2	Gm5860	chr4:82065430-82102808	0.572347	3.82766	-2.7415	<0.01	4 w vs. 0 w
ENSMUSG00000099411.1	2310015D24Rik	chr16:13514130-13521107	0.757382	4.27883	-2.49812	<0.01	4 w vs. 0 w
ENSMUSG00000087523.1	Gm12319	chr11:70654740-70656493	6.08675	149.492	-4.61826	<0.01	4 w vs. 0 w
ENSMUSG00000087410.7	2310065F04Rik	chr11:67112460-67120080	1.82899	24.5852	-3.74867	<0.01	8 w vs. 0 w
ENSMUSG00000000031.15	H19	chr7:142575528-142578143	2713.04	724.059	1.90573	<0.01	8 w vs. 0 w
ENSMUSG00000086503.3	Xist	chrX:103414466-103483217	31.4763	16.7478	0.910298	<0.01	8 w vs. 0 w
ENSMUSG00000085022.2	Gm5860	chr4:82065430-82102808	0.390679	3.82766	-3.29241	<0.01	8 w vs. 0 w
ENSMUSG00000099411.1	2310015D24Rik	chr16:13514130-13521107	1.15894	4.27883	-1.8844	<0.01	8 w vs. 0 w

*FPKM, fragments per kilobase of transcript per million fragments mapped; CON, control; INJ, injured; w, weeks.*

**Table 3:** The top 5 DE miRNAs at each time point.

miRNA	INJ read count	CON read count	Log2 fold change	*p*-value	Time
mmu-miR-21a-5p	233722.2	42418.82	2.3919	<0.01	1 w vs. 0 w
mmu-miR-142a-3p	410.3282	32.12056	3.3716	<0.01	1 w vs. 0 w
mmu-miR-142b	410.3282	32.12056	3.3716	<0.01	1 w vs. 0 w
mmu-miR-34c-5p	373.0039	27.33712	3.2644	<0.01	1 w vs. 0 w
mmu-miR-30e-3p	1164.2	2880.07	-1.2791	<0.01	1 w vs. 0 w
mmu-miR-21a-5p	237805.8	39936.37	2.5372	<0.01	2 w vs. 0 w
mmu-miR-7689-3p	111.2568	5.431634	3.7359	<0.01	2 w vs. 0 w
mmu-miR-208b-3p	325.846	27.52433	3.1762	<0.01	2 w vs. 0 w
mmu-miR-34c-5p	864.5815	25.60086	3.9759	<0.01	2 w vs. 0 w
mmu-miR-206-3p	376223.3	49594.49	2.6757	<0.01	2 w vs. 0 w
mmu-miR-34c-5p	1087.212	25.12119	4.9949	<0.01	4 w vs. 0 w
mmu-miR-1b-5p	1867501	5567081	-1.5628	<0.01	4 w vs. 0 w
mmu-miR-1a-3p	1870685	5572373	-1.5617	<0.01	4 w vs. 0 w
mmu-miR-7689-3p	164.5313	5.302219	4.515	<0.01	4 w vs. 0 w
mmu-miR-142a-3p	590.116	29.60037	3.9692	<0.01	4 w vs. 0 w
mmu-miR-214-5p	1022.621	198.5749	2.3336	<0.01	8 w vs. 0 w
mmu-miR-21a-5p	180822.3	37585.44	2.2365	<0.01	8 w vs. 0 w
mmu-miR-34c-5p	1057.082	24.20966	5.0399	<0.01	8 w vs. 0 w
mmu-miR-152-5p	296.5516	67.70904	2.1022	<0.01	8 w vs. 0 w
mmu-miR-208b-3p	792.2544	25.86505	4.5597	<0.01	8 w vs. 0 w

*CON, control; INJ, injured; w, weeks.*

**Table 4:** The top 5 DE circRNAs at each time point.

ID	INJ read count	CON read count	Log2 fold change	*p*-value	Time
novel_circ_0001810	201.7842	0	9.4785	<0.01	1 w vs. 0 w
novel_circ_0000090	173.8132	32.37428	2.4035	<0.01	1 w vs. 0 w
mmu_circ_0000213	322.68	78.53308	2.0247	<0.01	1 w vs. 0 w
novel_circ_0001811	881.5623	3755.757	-2.073	<0.01	1 w vs. 0 w
novel_circ_0011555	36.05462	175.3544	-2.2561	<0.01	1 w vs. 0 w
novel_circ_0015731	123.8905	635.3793	-2.3398	<0.01	2 w vs. 0 w
novel_circ_0001814	105.8517	0	8.7588	<0.01	2 w vs. 0 w
mmu_circ_0000474	22.72668	137.7106	-2.5687	<0.01	2 w vs. 0 w
novel_circ_0011555	26.52003	171.8074	-2.6377	<0.01	2 w vs. 0 w
novel_circ_0001680	59.58978	0	8.0033	<0.01	2 w vs. 0 w
novel_circ_0001717	0	587.2385	-10.855	<0.01	4 w vs. 0 w
novel_circ_0012758	2.117808	56.62443	-4.5687	<0.01	4 w vs. 0 w
mmu_circ_0000474	15.81561	135.3088	-3.0377	<0.01	4 w vs. 0 w
novel_circ_0001680	40.92397	0	7.6821	<0.01	4 w vs. 0 w
novel_circ_0015731	127.1867	623.1919	-2.2605	<0.01	4 w vs. 0 w
novel_circ_0001703	3458.893	0	13.313	<0.01	8 w vs. 0 w
novel_circ_0001774	0	1920.471	-12.304	<0.01	8 w vs. 0 w
novel_circ_0001794	128.24	1178.289	-3.1783	<0.01	8 w vs. 0 w
novel_circ_0001753	0	1204.574	-11.698	<0.01	8 w vs. 0 w
novel_circ_0001812	166.596	1548.646	-3.1936	<0.01	8 w vs. 0 w

*CON, control; INJ, injured; w, weeks.*

**Table 5:** The top 5 DE mRNAs at each time point.

Gene id	Gene name	Gene location	INJ FPKM	CON FPKM	Log2 fold change	*p*-value	Time
ENSMUSG00000049988	Lrrc25	chr8:70616843-70620850	3.78647	1.12963	1.74501	< 0.01	1 w vs. 0 w
ENSMUSG00000021904	Sema3g	chr14:31217859-31230350	2.97757	8.06068	-1.43676	<0.01	1 w vs. 0 w
ENSMUSG00000026384	Ptpn4	chr1:119652466-119837613	2.46588	7.5172	-1.60809	<0.01	1 w vs. 0 w
ENSMUSG00000033124	Atg9a	chr1:75180859-75210813	14.7636	36.9205	-1.32238	<0.01	1 w vs. 0 w
ENSMUSG00000030621	Me3	chr7:89632096-89854359	6.464	29.6673	-2.19838	<0.01	1 w vs. 0 w
ENSMUSG00000021904	Sema3g	chr14:31217859-31230350	3.85319	8.06068	-1.06485	<0.01	2 w vs. 0 w
ENSMUSG00000026384	Ptpn4	chr1:119652466-119837613	2.69564	7.5172	-1.47956	<0.01	2 w vs. 0 w
ENSMUSG00000030621	Me3	chr7:89632096-89854359	7.65026	29.6673	-1.95529	<0.01	2 w vs. 0 w
ENSMUSG00000010358	Ifi35	chr11:101448406-101458698	20.8814	9.68316	1.10867	<0.01	2 w vs. 0 w
ENSMUSG00000090877	Hspa1b	chr17:34956435-34959238	13.6025	5.57441	1.28698	<0.01	2 w vs. 0 w
ENSMUSG00000021904	Sema3g	chr14:31217859-31230350	2.35854	8.06068	-1.77301	<0.01	4 w vs. 0 w
ENSMUSG00000033124	Atg9a	chr1:75180859-75210813	14.5655	36.9205	-1.34186	<0.01	4 w vs. 0 w
ENSMUSG00000030621	Me3	chr7:89632096-89854359	5.32634	29.6673	-2.47766	<0.01	4 w vs. 0 w
ENSMUSG00000038936	Sccpdh	chr1:179668209-179687189	31.837	18.137	0.81177	<0.01	4 w vs. 0 w
ENSMUSG00000034842	Art3	chr5:92331826-92414628	20.9589	116.863	-2.47919	<0.01	4 w vs. 0 w
ENSMUSG00000021904	Sema3g	chr14:31217859-31230350	2.6483	8.06068	-1.60583	<0.01	8 w vs. 0 w
ENSMUSG00000033124	Atg9a	chr1:75180859-75210813	14.8694	36.9205	-1.31207	<0.01	8 w vs. 0 w
ENSMUSG00000030621	Me3	chr7:89632096-89854359	6.59861	29.6673	-2.16864	<0.01	8 w vs. 0 w
ENSMUSG00000090877	Hspa1b	chr17:34956435-34959238	10.9462	5.57441	0.973534	<0.01	8 w vs. 0 w
ENSMUSG00000034842	Art3	chr5:92331826-92414628	46.6576	116.863	-1.32464	<0.01	8 w vs. 0 w

*FPKM, fragments per kilobase of transcript per million fragments mapped; CON, control; INJ, injured; w, weeks.*

### Validation of ncRNA and mRNA Expression Using qPCR

**FIGURE 6:**
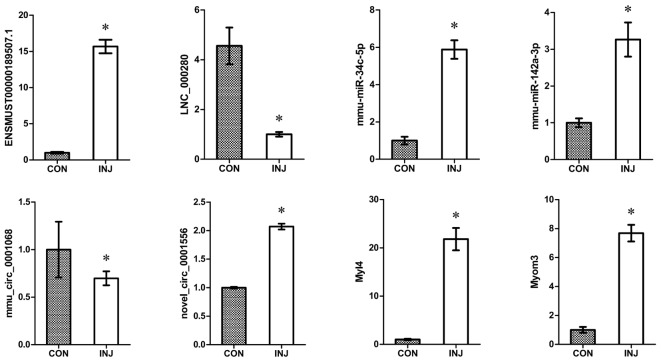
Validation of DE ncRNAs and mRNAs by qPCR. The results confirmed that the relative expression levels of ENSMUSG00000087523.1, LNC_000280, mmu-miR-34c-5p, mmu-miR-142a-3p, mmu_circ_0001068, novel_circ_0011051, Myl4, and Myom3 were significantly regulated after injury. ^∗^*P* < 0.05 compared to the control group. CON, control; INJ, injured.

We confirmed the accuracy of sequencing data for selected ncRNAs and mRNAs (ENSMUSG00000087523.1, LNC_000280, mmu-miR-34c-5p, mmu-miR-142a-3p, mmu_circ_0001068, novel_circ_0011051, Myl4, and Myom3) that were DE after injury using qPCR. The mRNA validation results were consistent with the RNA-seq data (**Figure 6**). These convincing sequencing data can be used as the basis for future research.

### Functional Annotation Based on GO

**FIGURE 7:**
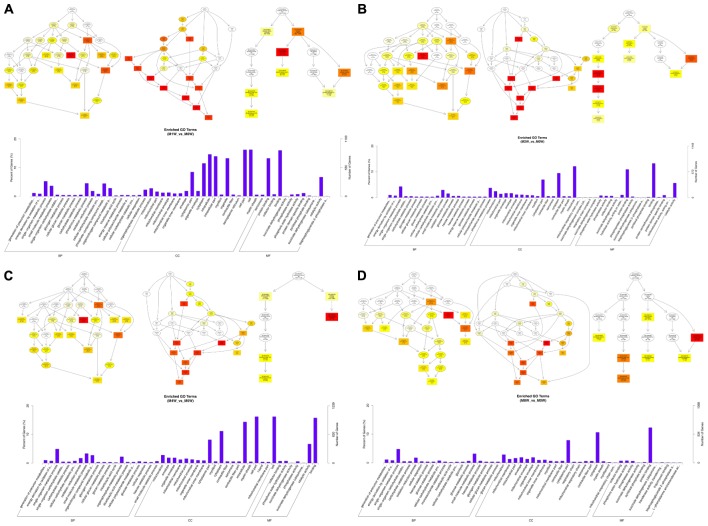
Gene Ontology (GO) analysis of lncRNAs at each time point. DAG showing the significant BP, CC, and MF. Histograms displaying the numbers of genes in each GO term (**A**: 1 week vs. 0 week, **B**: 2 weeks vs. 0 week, **C**: 4 weeks vs. 0 week, **D**: 8 weeks vs. 0 week).

**FIGURE 8:**
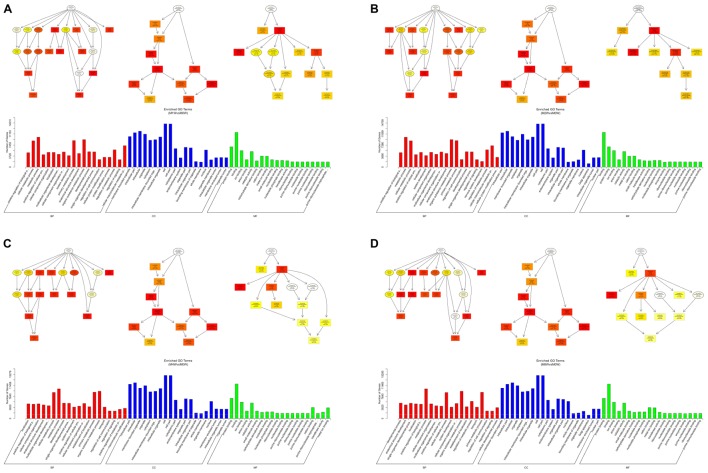
Gene Ontology analysis of miRNAs at each time point. DAG showing the significant BP, CC, and MF. Histograms displaying the numbers of genes for each GO term (**A**: 1 week vs. 0 week, **B**: 2 weeks vs. 0 week, **C**: 4 weeks vs. 0 week, **D**: 8 weeks vs. 0 week).

**FIGURE 9:**
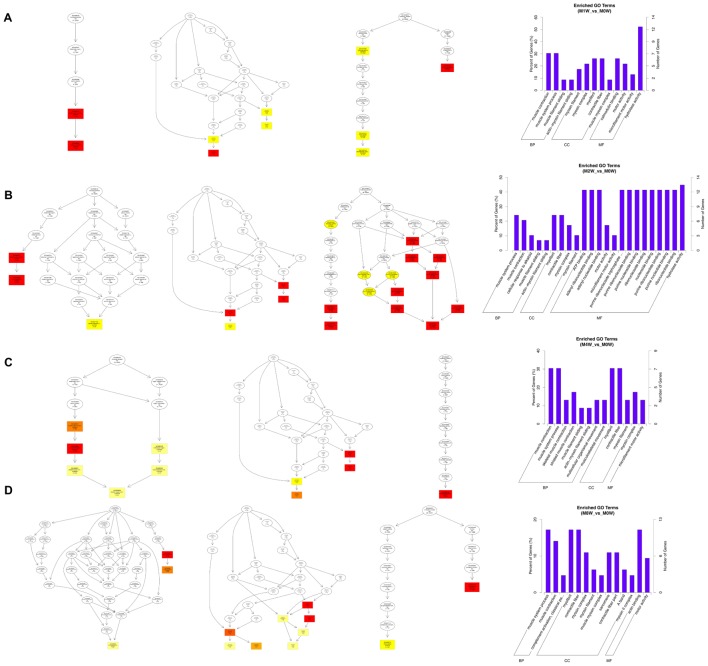
Gene Ontology analysis of circRNAs at each time point. DAG showing the significant BP, CC, and MF. Histograms displaying the numbers of genes for each GO term (**A**: 1 week vs. 0 week, **B**: 2 weeks vs. 0 week, **C**: 4 weeks vs. 0 week, **D**: 8 weeks vs. 0 week).

**FIGURE 10:**
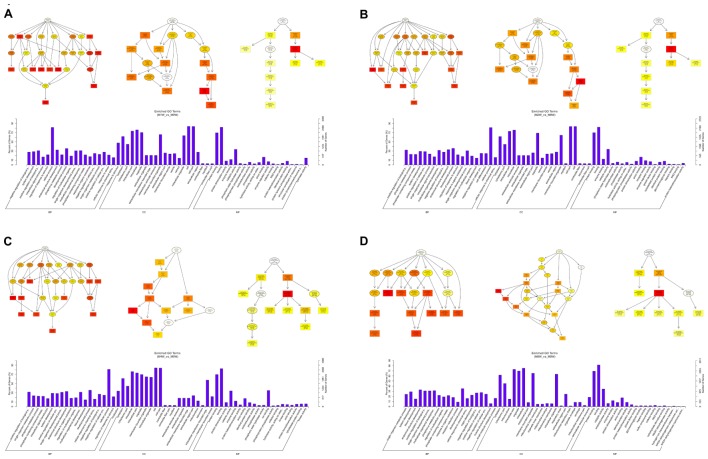
Gene Ontology analysis of mRNAs at each time point. DAG showing the significant BP, CC, and MF. Histograms displaying the numbers of genes for each GO term (**A**: 1 week vs. 0 week, **B**: 2 weeks vs. 0 week, **C**: 4 weeks vs. 0 week, **D**: 8 weeks vs. 0 week).

To predict the functions of the mRNAs in PNI, genes with absolute correlation values >0.95 were selected and underwent GO and KEGG pathway analysis. The GO enrichment analysis results for the DE lncRNAs, miRNAs, circRNAs, and mRNAs are shown in **Figures 7–10**, respectively. Directed acyclic graphs (DAGs) are used to display the GO results. In these graphs, the relevance of the DE genes is illustrated by the branching pattern from top to bottom. Using the master node, which represents the enrichment degree by color depth, the top 10 GO enrichment analysis results, including their relationships, are shown together in the DAG. The GO analysis was performed for three major groups of RNAs involved in biological process (BP), cellular component (CC), and molecular function (MF). A histogram was used to display the numbers and percentages of genes significantly enriched for each GO term at each time point. The GO analysis details are summarized below.

Based on the co-expressed genes with the DE lncRNAs, the most significantly enriched BP involved the generation of precursor metabolites and energy at 1, 2, 4, and 8 weeks after the injury. The most involved BP were related to single-organism metabolic processes at 1, 2, 4, and 8 weeks after the injury. The most significantly enriched CC was the mitochondrion at 1, 2, 4, and 8 weeks after injury. The most involved CC were the cell at 1 and 4 weeks after injury, intraCCs at 2 weeks after the injury, and the cytoplasm at 8 weeks after the injury. The most significantly enriched MF were protein binding at 1 week after the injury, phosphoric ester hydrolase activity at 2 weeks after the injury, and cofactor binding at 4 and 8 weeks after injury. The most involved MF were binding at 1, 2, and 4 weeks after injury, and protein binding at 8 weeks after injury (**Figure 7**).

Based on the target genes of the DE miRNAs, the most significantly enriched BP were the positive regulation of biological processes at 1 and 2 weeks after injury, localization at 4 weeks after injury, and developmental processes at 8 weeks after injury. The most involved BP were metabolic processes at 1, 2, 4, and 8 weeks after the injury. The most significantly enriched CC were membrane-bounded organelles at 1 and 8 weeks after injury, and intracellular componenets at 2 and 4 weeks after injury. The most involved CC was the cell at 1, 2, 4, and 8 weeks after injury. The most significantly enriched MF were protein binding at 1, 4, and 8 weeks after injury, and binding at 2 weeks after injury. The most involved MF was binding at 1, 2, 4, and 8 weeks after injury (**Figure 8**).

Based on the target genes of the DE circRNAs, the most significantly enriched BP were muscle contraction at 1 and 4 weeks after injury, and muscle system processes at 2 and 8 weeks after the injury. The most involved BP were muscle contraction at 1 and 4 weeks after injury, and muscle system processes at 2 and 8 weeks after injury. The most significantly enriched CC were myosin filaments at 1 week after injury, and myofibrils at 2, 4, and 8 weeks after injury. The most involved CC were myofibrils at 1, 2, 4, and 8 weeks after injury. The most significantly enriched MF were calmodulin binding at 1 week after the injury, adenosine triphosphate binding at 2 weeks after injury, microfilament motor activity at 4 weeks after injury, and actin binding at 8 weeks after the injury. The most involved MF were hydrolase activity at 1 and 2 weeks after injury, adenosine triphosphate binding at 4 weeks after injury, and cytoskeletal protein binding at 8 weeks after injury (**Figure 9**).

Based on the target genes of the DE mRNAs, the most significantly enriched BP were the negative regulation of biological processes at 1 week after injury, positive regulation of biological processes at 2 and 4 weeks after injury, and single-organism metabolic processes at 8 weeks after injury. The most involved BP were cellular processes at 1, 2, and 4 weeks after injury, and developmental processes at 8 weeks after the injury. The most significantly enriched CC was the cytoplasm at 1, 2, 4, and 8 weeks after injury. The most involved CC were the cell at 1, 2, and 4 weeks, and intraCCs at 8 weeks after the injury. The most significantly enriched MF was protein binding at 1, 2, 4, and 8 weeks after surgery. The most involved MF was binding at 1, 2, 4, and 8 weeks (**Figure 10**).

### Pathway Prediction Based on KEGG

**FIGURE 11:**
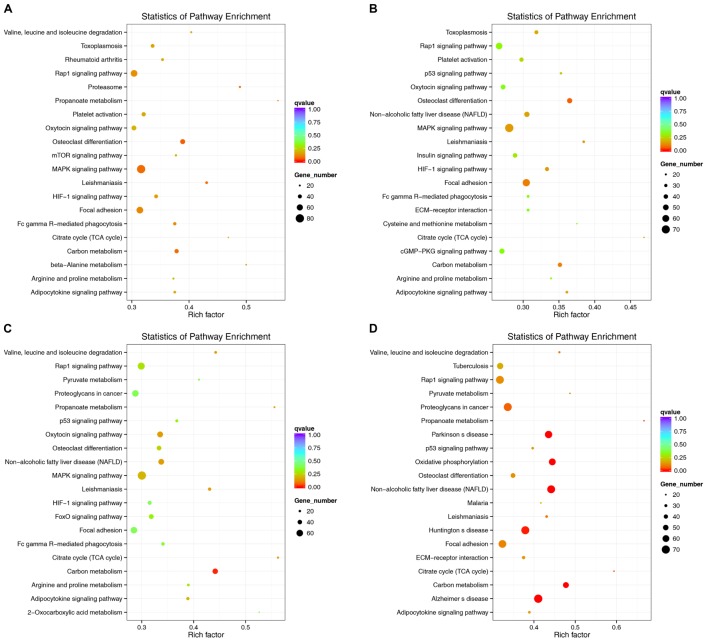
Kyoko Encyclopedia of Genes and Genomes (KEGG) pathway analysis at each time point. Scatterplots displaying the lncRNA-miRNA-mRNA enriched pathways in KEGG. (**A**: 1 week vs. 0 week, **B**: 2 weeks vs. 0 week, **C**: 4 weeks vs. 0 week, **D**: 8 weeks vs. 0 week).

lncRNAs have extensive regulatory functions that can directly regulate the structure of DNA and the transcription and translation of RNA. They can also inhibit target gene regulation by miRNAs to indirectly regulate gene expression. An enriched scatter diagram was used to display the KEGG enrichment analysis results for the intersection of genes co-localized and co-expressed with the DE lncRNAs, target genes of the DE miRNAs, and predicted mRNAs. The degree of KEGG enrichment was reflected by the Rich factor, the *Q*-value, and the number of genes. Based on the KEGG results, the top 5 significantly involved pathways in the pathogenesis of denervated muscle atrophy were osteoclast differentiation, MAPK signaling, carbon metabolism, the proteasome, and leishmaniasis 1 week after the injury; osteoclast differentiation, focal adhesion, carbon metabolism, MAPK signaling, and hypoxia-induced factor-1 signaling 2 weeks after the injury; carbon metabolism, citric acid cycle, leishmaniasis, non-alcoholic fatty liver disease, and oxytocin signaling 4 weeks after injury; and non-alcoholic fatty liver disease, carbon metabolism, Parkinson’s disease, oxidative phosphorylation, and Alzheimer’s disease 8 weeks after the injury (**Figure 11**). The MAPK signaling pathway was the most involved pathway in all groups based on the number of participating genes. The genes involved in the MAPK signaling pathway might play crucial roles in denervated muscle atrophy and are thus worthy of further research. These results illustrate the regulatory relationships between ncRNAs and mRNAs in the mechanisms underlying denervated muscle atrophy after PNI. Further in-depth studies by our team will be forthcoming.

## Discussion

Peripheral nerve injury, especially that involving long nerve segments, may result in poor functional recovery such as progressive skeletal muscle atrophy, which occurs until axons regenerate into the target tissue. Previous studies have shown that some ncRNAs and mRNAs can regulate the progress of nerve regeneration in response to muscle atrophy (Pan et al., 2015; Huang et al., 2016). This is the first study to investigate the expression profile of the whole transcriptome, including ncRNAs and mRNAs, during skeletal muscle atrophy after PNI using sequencing analysis. We also used GO and KEGG pathway analyses to predict the potential functions and mechanisms of the DE RNAs. Our results support the opinion that ncRNAs play key roles in denervated muscle atrophy pathogenesis, and may highlight a potential therapeutic target for the treatment of this condition.

Peripheral nerve injury is a common clinical disease. Incomplete nerve regeneration leads to poor recovery, such as loss of limb movement. Long periods of denervation lead to several progressive degenerative processes, resulting in atrophy of the denervated muscle. In the absence of axonal regeneration, the target muscles lose volume and undergo fibrosis. In addition, the number of myofibrils is reduced and they decrease in size, while the number of receptive motor endplates is decreased (Carlson et al., 1996; Ma et al., 2011). Previous studies from our group have been performed to accelerate axonal regeneration using experimental therapies (Wei et al., 2009; Kou et al., 2013). Today, the ideal therapy would also enable the maintenance of muscle functions during the regeneration process. Kobayashi et al. (1997) have reported significant changes in both muscle mass and whole motor function when the nerve regeneration interval is longer than 1 month. Thus, therapeutic prevention of muscle atrophy following PNI may be beneficial in these cases. Recent studies support the opinion that early intervention is appropriate for denervated muscles when reinnervation is delayed, or in the worst case, when there is no opportunity for reinnervation (Gao et al., 2005; Deshpande et al., 2006). Therefore, the identification of molecular mechanisms for the prevention of skeletal muscle atrophy is necessary.

ncRNAs have been shown to play important roles in the regulation of specific target mRNAs and proteins in many diseases, including muscle atrophy (Crist and Buckingham, 2010; Russell et al., 2013). Eisenberg et al. (2007) have identified 185 miRNAs that are significantly regulated in 10 major muscular disorders. Greco et al. (2009) have reported that some miRNAs related to regeneration and degeneration were modified in both patients with muscular dystrophy and in mouse models. The above studies indicate the important role of miRNAs in pathophysiological pathways in muscle atrophy. However, no studies have investigated ncRNAs, which include lncRNAs and circRNAs, during denervated muscle atrophy. We utilized RNA-seq to detect and further analyze the biological roles of DE ncRNAs and mRNAs in denervated gastrocnemius muscles. Six-hundred and sixty-four lncRNAs, 671 miRNAs, 236 circRNAs, and 12,768 mRNAs were significantly DE during muscle atrophy. Our findings regarding ncRNAs may provide a theoretical basis for the study of the mechanisms underlying denervated muscle atrophy. Due to the special regulatory functions of lncRNAs, it is worth mentioning that 73 DE lncRNAs were detected during the progression of atrophy after PNI. These lncRNAs should be considered potential therapeutic targets in further studies.

Gene Ontology analysis is currently the main bioinformatics tool used to unify the representation and product attributes of genes (Altshuler et al., 2008). It has been confirmed that GO terms and GO annotations can be used to predict gene functions and trends (Du et al., 2016). The KEGG pathway database is currently widely used as an enrichment analysis platform for gene functions due to the high levels of functional information that can be used for systematic analysis (Camon et al., 2004). We investigated gene functions and pathways related to the DE ncRNAs and mRNAs in the gastrocnemius muscle after PNI using GO and KEGG analyses. Our results indicated that the most significantly involved pathway in the pathogenesis of denervated muscle atrophy was the MAPK signaling pathway. Other pathways involved in denervated muscle atrophy, such as carbon metabolism, the proteasome, focal adhesion, the citric acid cycle, and oxidative phosphorylation are all fundamental biological processes present in all organisms. The results of the GO and KEGG analyses support investigation of the underlying mechanisms of denervated muscle atrophy after PNI. The accuracy of the sequencing data as the basis for future research was confirmed. It is well known that higher biological replicate numbers can minimize the false positive rate in RNA-seq results. A limitation of our study was that we tested three samples in parallel in each group to obtain the minimum number of biological replicates. However, we strictly controlled other variables (animal genetic background, feeding conditions, age, sex, and external shape). Furthermore, the correlation analysis results suggested that the individual differences in the samples were small in each group.

Our study is the first to investigate the expression profile of the whole transcriptome in denervated muscle atrophy using innovative RNA-seq analysis. Here we provide a novel bioinformatics platform for the study of the roles of ncRNAs and mRNAs in denervated muscle atrophy, which may be benefit future research. This study supports the opinion that ncRNAs, and especially lncRNAs, may have interactions and regulatory relationships with their co-localized and co-expressed genes, which may in turn affect the pathogenesis of denervated muscle atrophy. However, the regulatory roles of ncRNAs in the pathogenesis of denervated muscle atrophy are complicated and largely unknown. Our team will further investigate the related genes and corresponding signaling pathways for the predicted ncRNAs and mRNAs in a future study to elucidate the mechanisms underlying denervated muscle atrophy.

## Ethics Statement

The study was approved by the Animal Experiment Ethics Committee of Peking University People’s Hospital. All of the procedures were performed in accordance with the relevant guidelines and regulations.

## Author Contributions

BJ and XY designed the study. JW performed the experiments and wrote the manuscript. PZ contributed to the analysis of all data. All authors reviewed the manuscript.

## Conflict of Interest Statement

The authors declare that the research was conducted in the absence of any commercial or financial relationships that could be construed as a potential conflict of interest.
